# Institutional trust, scientific literacy, and information sources: What factors determine people's attitudes toward COVID-19 vaccines of different origins in China?

**DOI:** 10.3389/fpubh.2023.1092425

**Published:** 2023-02-20

**Authors:** Yanyu Ye, Zhenhua Su, Chunyu Shi

**Affiliations:** ^1^Department of Public Administration, School of Law, Hangzhou City University, Hangzhou, China; ^2^Department of International Culture, College of Media and International Culture, Zhejiang University, Hangzhou, China; ^3^Department of Public Administration, School of Public Management, Zhejiang Gongshang University, Hangzhou, China

**Keywords:** COVID-19 vaccines, vaccine attitudes, institutional trust, scientific literacy, information sources

## Abstract

**Objective:**

This study aimed to investigate the different attitudes of Chinese residents toward COVID-19 vaccines produced in China and the United States in an emergency context, and then explored possible explanations for these different attitudes.

**Methods:**

Using data collected online in May 2021, we compared Chinese citizens' attitudes toward vaccines originating from China and the US and then adopted ordered logistic models to examine how trust in institutions, scientific literacy, and information sources influence their attitudes toward different vaccines.

**Results:**

A total of 2038 respondents completed the survey. Participants reported very different levels of trust in Chinese and American vaccines. The main finding of this paper is that individuals who trust in Chinese institutions, especially those who trust in domestic scientists, typically feel encouraged to also place their trust in domestic vaccines and to distrust those from the US. These individuals' higher evaluation of Chinese government performance makes them more willing to vaccinate with domestic vaccines and less likely to seek US vaccines. Levels of scientific literacy, furthermore, seem to have little influence on attitudes toward different vaccines. Meanwhile, respondents who acquire health information from biomedical journals are more likely to hold a positive view of US vaccines, and these individuals contribute to bridging the gap between levels of trust in Chinese and US vaccines.

**Conclusions:**

In contrast with previous findings about Chinese attitudes toward imported vaccines, our respondents are more convinced of the safety and effectiveness of domestic vaccines than of US ones. This trust gap does not arise out of actual disparity in the quality and safety of the different vaccines *per se*. Instead, it is a cognition concern that is closely bound up with individuals' trust in domestic institutions. People's attitudes toward vaccines of different origins in an emergency context are more influenced by socio-political beliefs than by concern with objective information and knowledge.

## 1. Introduction

Since the world-wide outbreak of the novel Coronavirus disease (COVID-19) in the spring of 2020, vaccination has been considered as one of the most effective public health tools to combat the pandemic. Various vaccines have been rapidly developed and successively become available in many countries since late 2020 (see https://ourworldindata.org/covid-vaccinations). Up to May 2022, the WHO has validated 11 COVID-19 vaccines from the United States, China, Europe, and India for global emergency use based on their reliability, safety, and efficacity. However, the availability of effective and safe vaccines does not constitute a sufficient condition for successfully combatting a pandemic, which is dependent on people's trust in vaccines and willingness to be vaccinated (1). Scholars from different countries and cultures have showed the persistence of vaccine hesitancy and distrust over time (2, 3), even during periods of severe outbreak such as the COVID-19 pandemic (4, 5). Moreover, different vaccine products have been trusted to greater or lesser extents depending on specific societal and cultural context (6, 7). For example, a national survey conducted in Poland that aimed to assess public trust in different types of COVID-19 vaccine found that the Pfizer-BioNTech, Moderna, and CVnCoV vaccines were highly trusted, while the Sputnik vaccine was not (8). In contrast, findings from a study conducted in Sri Lanka indicate that individuals have a higher level of trust in the Sputnik vaccine compared to vaccines produced by Moderna and Pfizer (9).

The COVID-19 vaccination campaign in China began officially on December 15, 2020. At this point, the pandemic was temporarily under control, but the situation was severe in other countries such as the Unites states, Britain, and France, which were reporting tens of thousands of infections and hundreds of deaths each day. In this context, vaccination was considered as necessary and the most effective strategy to combat the pandemic in public discourse. Unlike in the US and some European countries, the COVID-19 vaccination is voluntary in China, but different levels of the government have carried out an extensive social mobilization: besides the use of media propaganda, official leaders and medical experts took the lead in vaccination demonstration, and the vaccination is totally free and available in each community and village. The vaccines that Chinese people receive are nearly all domestically produced, although the country has also imported 100 million dozes of Pfizer-BioNTech vaccines as reserves. Compared to mRNA vaccines such as Pfizer-BioNTech and Moderna made with modern technology, Chinese COVID-19 vaccines such as Sinovac and Sinopharm are inactivated vaccines and made with traditional methods.

Given the findings of previous studies showing that Chinese people trust more or at least equally imported vaccines compared to domestic vaccines for protecting against measles-mumps-rubella, HPV, and other diseases (10, 11), what might be their perceptions of COVID-19 vaccines from China and other countries? Compared to the former vaccines, with which people are quite familiar, those for COVID-19 are totally new and have been developed in a very short timeframe. Their rapid development caused concerns around efficacity and safety (12): Were these vaccines equally effective and safe? Or did some of them contain more substantial side effects? Lay people are unable to provide answers to these questions because they lack adequate expertise. Even though scientific studies have reported that fully vaccinated recipients of each of the afore-mentioned COVID-19 vaccines all have efficient immunogenicity profiles (13, 14), people's attitudes toward these various vaccines might nevertheless differ, as there's a lack of long-term, consistent, and compelling evidence-based information for their effectiveness and safety, especially regarding potential adverse effects.

Vaccine trust issues are usually thought to be context-specific (15). Previous studies have shown that people's attitudes toward vaccines are affected by both objective knowledge (which assumes that the public is rational) and psychological factors such as socio-political beliefs (which assumes that the public is emotional) (5, 16, 17), but it is unclear how these rational and emotional factors influence people's attitudes in specific contexts such as an emergency situation. An emergency situation is marked by three essential characteristics (18, 19): (1) The threat from a risk is immediate and substantial; (2) People need to make a key decision in a limited time; and (3) Reliable information available for decision making is scarce and insufficient.

The purpose of this study is to examine whether objective knowledge or socio-political beliefs determined Chinese attitudes toward COVID-19 vaccines during the global outbreak period. Objective knowledge is related to individuals' scientific literacy, while socio-political belief involves institutional trust. Given the central importance of channels of information in shaping individuals' perceptions of vaccines (20–22), we also examine this as a key independent factors, in addition to scientific literacy and institutional trust. In order to better explore the nuances of these different influencing factors in shaping people's attitudes, we investigate the question by comparing the differing attitudes of Chinese people toward COVID-19 vaccines from the United States and China.

## 2. Methods

### 2.1. Data source

The data for this study were obtained from a national survey conducted online in May of 2021, when the Chinese government was pushing COVID-19 vaccination to all people. The survey was aimed to investigate public's attitudes toward the newly developed vaccines. We entrusted a Shanghai-based research company, Diaoyanba, to carry out the survey. The potential participants were selected from the sample pool of the company by stratified random sampling method, to make sure that the regional (provincial) geographic locations, age cohorts, and gender distribution of the sample match the demographic data of the 2019 China Statistical. We invited 8,000 participants to fill out the questionnaire by email and informed them that they could withdraw from the study at any time if they felt uncomfortable. Those who completed the questionnaire would receive a small monetary reward of 18 yuan (~$2.59). Finally, 4,533 participants responded to our survey, with 2,495 give up halfway and 2,038 complete the survey. The study population consisted of 51.28% males and 48.72% females, with representation from 31 provincial regions across China. Further demographic information can be found in Table 1.

**Table 1:** The definition of variables.

**Variables**	**Variable definition**	**Mean**	**Std. dev**.
**Dependent variables**			
**Vaccine assessment**			
The domestic inactivated vaccines are safer	Ordinal variable; Absolutely disagree = 1, Quite agree = 5	4.690	0.618
The domestic inactivated vaccines are more effective	Ordinal variable; Absolutely disagree = 1, Quite agree = 5	4.629	0.662
The US mRNA vaccines are safer	Ordinal variable; Absolutely disagree = 1, Quite agree = 5	2.159	1.291
The US mRNA vaccines are more effective	Ordinal variable; Absolutely disagree = 1, Quite agree = 5	2.134	1.288
**Vaccination preference**			
Even if imported COVID−19 vaccines are available, I will give priority to taking domestic vaccines	Ordinal variable; Absolutely disagree = 1, Quite agree = 5	4.693	0.778
If possible, I will give priority to taking COVID−19 vaccines developed in the US	Ordinal variable; Absolutely disagree = 1, Quite agree = 5	1.636	0.873
**Independent variables**			
**Institutional trust**			
Government performance perception	Factor analysis	0	1
Trust in Chinese medical experts	Ordinal variable; Absolutely distrust = 1, Quite trust = 10	9.376	1.224
**Scientific literacy**			
Scientific literacy	Continuous variable	2.730	1.662
**Information sources**			
Medical experts	Ordinal variable, 1–5	3.028	0.996
Traditional media	Ordinal variable, 1–5	3.156	1.065
Biomedical journal	Ordinal variable, 1–5	2.693	1.067
Social media	Ordinal variable, 1–5	2.884	1.119
**Control variables**			
**Risk perception**			
Perceived COVID−19 infection likelihood	Ordinal variable, 1–5	2.043	1.305
Perceived severity of COVID−19	Ordinal variable, 1–5	3.859	1.406
**Demographic variables**			
Age	**Ordinal variable**		
	“18–29” = 1	21.74%	
	“30–39” = 2	25.86%	
	“40–49” = 3	26.59%	
	“50–59” = 4	25.81%	
Gender	**Dummy variable**		
	Male = 0,	51.28%	
	Female = 1	48.72%	
Education	**Ordinal variable**		
	Junior high school and below = 1	12.41%	
	Senior high school = 2	17.47%	
	Associate degree = 3	33.95%	
	Bachelor's degree = 4	33.76%	
	Master's degree and above = 5	2.4%	
Monthly income	**Ordinal variable**		
	3,000 and below = 1	25.07%	
	3,001–5,000 = 2	36.01%	
	5,001–10,000 = 3	30.57%	
	10,001–20,000 = 4	6.97%	
	20,001 and above = 5	1.18%	

### 2.2. Research design

First, we compared respondents' attitudes toward domestic COVID-19 vaccines to their attitudes toward vaccines produced in the US, and we examined their vaccination preference.

Second, we adopted the ordered logistic regression model to examine how institutional trust, scientific literacy, and information sources influence public perceptions of vaccine quality and people's preference for either Chinese- or US-made vaccines.

Third, we explored the correlation between perceived vaccine quality and vaccine preference.

### 2.3. Measures

#### 2.3.1. Dependent variables

To measure individuals' attitudes toward domestic- and US-produced COVID-19 vaccines, we selected three pairs of variables. Fist, respondents were asked to assess the perceived quality of domestic vaccines from two aspects, measured by the questions “To what extent do you agree that the domestic vaccines are safer” and “To what extend do you agree that the domestic vaccines are more effective.” Second, respondents were asked to judge the relative quality of vaccines developed by China and the United States, measured by the questions “To what extent do you agree that the US vaccines are safer than domestic vaccines” And “To what extend do you agree that the US vaccines are more effective than domestic vaccines.” The third pair of dependent variables concerns individuals' vaccination preference, which was measured by two items: “Even if there are imported COVID-19 vaccines, I will still give priority to taking domestic vaccines;” and “If possible, I will give priority to taking COVID-19 vaccines developed in the US.” Respondents answered on a scale ranging from “1 (strongly disagree)” to “5 (totally agree).”

#### 2.3.2. Independent variables

##### 2.3.2.1. Institutional trust

Previous studies have indicated that vaccine hesitancy among members of the public is often linked to their negative perception of government performance in dealing with the crisis (2, 7). Infection rates and the reported number of COVID-19-related deaths in China were far less than in other countries. Since 2020, a discourse has been widely distributed through Chinese public media channels which affirms that the Chinese government's response to COVID-19 has been one of the most efficient in the world (23). Therefore, we hypothesize that Chinese people's confidence in domestic vaccines is closely associated with their trust in the domestic epidemic prevention institution (**RQ1**).

In this study, trust in the institution was measured by respondents' subjective evaluation of government performance in dealing with the epidemic. This involved measuring respondents' opinions about government performance in the following three dimensions: containing the spread of the virus; protecting citizens from the epidemic; and the recovery of economic activities. Respondents' answers were ranged from “1 (strongly disagree that the government performed well)” to “5 (strongly agree that the government performed well).” Due to high correlation among the three factors, we tried to solve the multicollinearity problem with factor analysis. The value of a KMO test is 0.705, and the result of a Bartlett test of sphericity is significant (*p*-value < 0.000), indicating that it is plausible to conduct factor analysis. After factor analysis, only one eigenvalue was higher than “1,” and thus we finally determined one common factor, which we termed the government performance factor.

In addition to trust in government performance, trust in domestic medical experts has also been considered an important predictor of institutional trust. In China, medical experts are quasi-civil servants who work under government management and supervision. They not only provide professional advice to government for health-related policy-making; they are also supposed to interpret government policies and discourse, both for members of the public and those in professional practices. Therefore, the public's trust in domestic medical experts constitutes another manifesting form of institutional trust. In the questionnaire, participants were asked to assess how reliable Chinese medical experts are, with 1 indicating “not at all trustworthy” and 10 indicating “very trustworthy.”

##### 2.3.2.2. Scientific literacy

Many previous studies have considered scientific literacy to be an important influencing factor in people's perception of the safety and effectiveness of vaccines (24–26), because the accumulation of scientific literacy can eventually help individuals to discern the quality of different types of vaccines (**RQ2**). In this study, scientific literacy is measured by seven statements, with participants asked to say whether each statement was true or false. The statements concerned knowledge of vaccines, viruses, and basic biological theories. They included: “Smallpox would not have been eradicated without the widespread use of vaccines;” “Vaccination does not increase the incidence of allergies;” “Coronaviruses can cause SARS and pneumonia, but cannot cause colds;” “Antibiotics (such as penicillin-streptomycin or cephalosporin) can kill viruses as well as bacteria;” “Mothers' genes can determine the gender of their children;” and “A person's genes may become altered if he/she eats genetically modified fruit.” One point is counted for each correct answer.

##### 2.3.2.3. Information sources

Respondents' sources of health information were also an important factor influencing people's attitudes toward vaccines (27–29). Generally speaking, individuals obtain health information mainly through the following four channels: (1) professional medical personnel, because they are considered as possessing professional knowledge and expertise, and therefore lay people often turn to them to obtain information about vaccines when their own knowledge is insufficient; (2) traditional media, including newspapers, television and radios, which followed up and reported on the latest situation during the outbreak period, constituting an important channel of epidemic-related information; (3) biomedical journals, in which a large number of novel Coronavirus-related articles were published, revealing the latest scientific research results; and (4) social media, which has become an important channel for information exchange in the internet era. Previous studies have examined the correlation between social media usage and users' attitude toward vaccines and vaccination (30). As it concerns everyone's safety and health, the epidemic has triggered abundant discussions online, and these have been an important source of health information for many people. This study will examine how different health information sources influenced the attitudes of members of the public toward vaccines of different origins (**RQ3)**. In the questionnaire, respondents were asked how often they acquire health information from medical personnel, traditional media, social media, and biomedical journals, with possible answers ranging from “1 (hardly ever)” to “5 (always).”

#### 2.3.3. Control variables

##### 2.3.3.1. Risk perception

Numerous studies have found that higher perception of risk from COVID-19 is likely to result in higher acceptance of vaccination (31–33). In this research, risk perception was measured by participants' responses to the following two statements: “I am likely to be infected by COVID-19;” and “COVID-19 is a major threat to my health.” Respondents answered on a scale ranging from “1 (strongly disagree)” to “5 (strongly agree).”

##### 2.3.3.2. Demographic variables

Numerous studies have found that individuals' demographic characteristics are important influencing factors on their attitude toward vaccination. For example, older people seem more favorable to vaccination as they are vulnerable to infectious diseases (34). In addition, socioeconomic status and gender can influence perceptions of health policy and thus in turn affect individuals' attitudes toward vaccines (35, 36). Accordingly, the control variables in this study focused on demographic characteristics such as age, gender, education, and monthly income of the respondent.

### 2.4. Statistical model

The purpose of this study is to test the effects of individuals' institutional trust, scientific literacy, and information sources on attitudes toward domestic COVID-19 vaccines and vaccines imported from the United States. Considering that the dependent variable is rated with a five-point Likert scale, this study employed ordered Logistic regression models. We adopted stata 14.0 to analyze the data and reported the coefficient and adjusted odds ratios with 95% CI in tables.

## 3. Results

### 3.1. Vaccine attitudes

**Figure 1:**
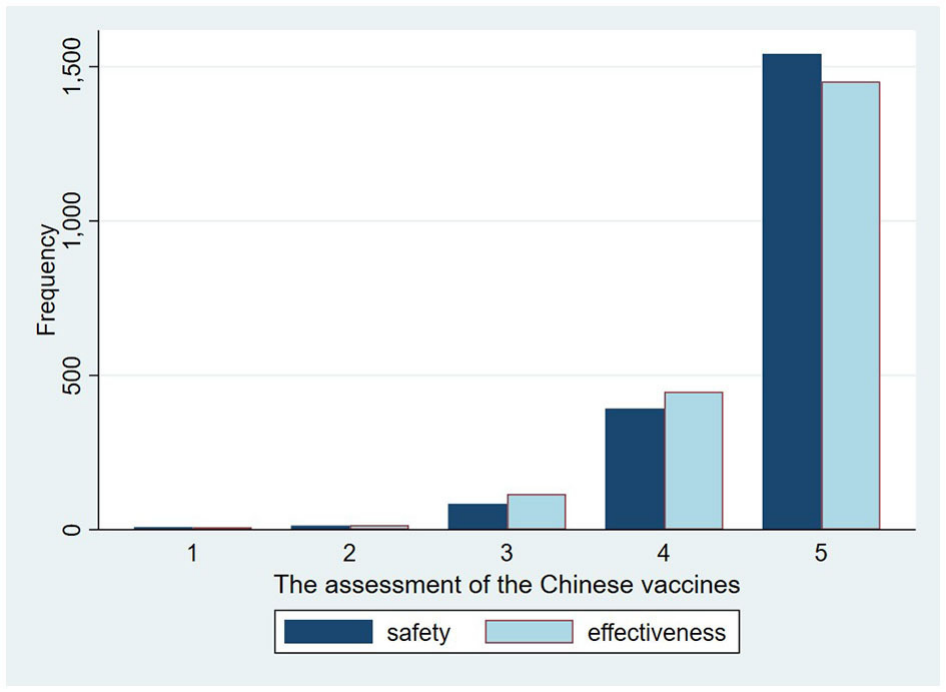
The perceived safety and effectiveness of the Chinese COVID-19 vaccines.

**Figure 2:**
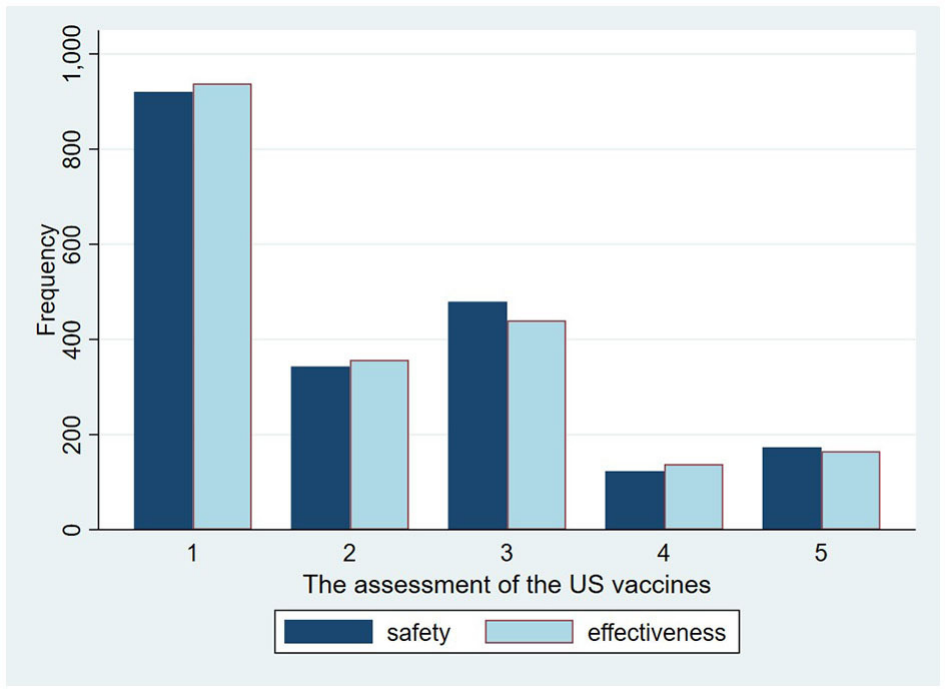
The perceived safety and effectiveness of the US COVID-19 vaccines.

**Figure 3:**
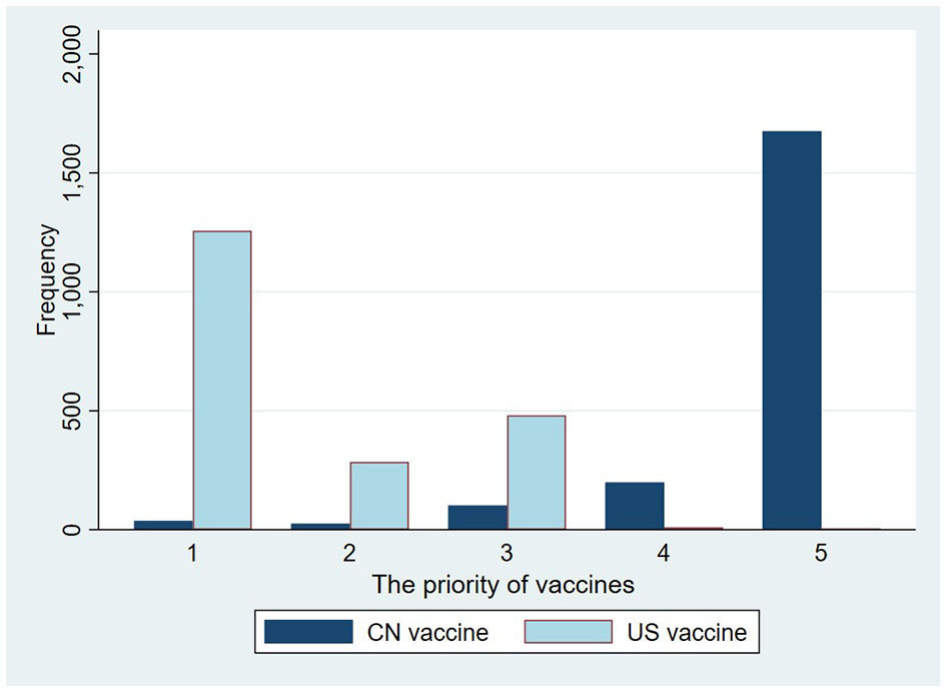
Respondents' preferences for the Chinese and American vaccines.

According to our results, there is a similar distribution and close relationship between the perceived safety and effectiveness of the same vaccines. According to our analysis results, more than 70% of respondents strongly agree that the domestic vaccines are safer or more effective than the vaccines from the US (Figure 1), while more than 60% of participants “strongly or somewhat” disagree that the safety and effectiveness of US vaccines are better than that of the domestic ones (Figure 2). As for vaccination preferences, 82.19% of respondents expressed strong willingness to receive the domestic vaccines, even if imported vaccines from the US were available (Figure 3). Nearly three quarters of participants “strongly or somewhat” disagree with the statement “I prefer to vaccinate with the COVID-19 vaccines developed in the US rather than with the domestic vaccines.” The distribution of respondents' answers indicates a clear difference between public attitudes toward Chinses and US vaccines, which is in contrast with the findings of previous studies, carried out before the global outbreak of COVID-19.

### 3.2. Influencing factors and vaccine attitudes

#### 3.2.1. Influencing factors and perceptions of vaccine quality

Our examination of the relationship between institutional trust, scientific literacy, and information usage revealed how they influence attitudes toward vaccines produced in China and the US. These results can be found in Tables 2, 3. The outcomes of the likelihood ratio test (LR test) showed that the Models 1–4 are all statistically significant when compared to the null model with no predictors. The classification accuracy (measured by the $R_{\text{count}}^{2}$ coefficient) was 72.27, 73.6, 44.7, and 45.1%. The Nagelkerke *R*^2^ showed that Models 1 and 2 can, respectively explaining 26.3 and 27.6% of the variation in the perceived safety and effectiveness of domestic vaccines, Models 3 and 4 can explain 10.0 and 11.1% of the variation in the perceived safety and effectiveness of the US vaccines.

**Table 2:** Regression results of attitudes toward domestic vaccines.

	**Safety (CN)**		**Effectiveness (CN)**	
	**(Model 1)**		**(Model 2)**	
	β_1_ **(SE)**	**Adjusted OR**_1_ **(95%CI)**	β_2_ **(SE)**	**Adjusted OR**_2_ **(95%CI)**
**Key variables**				
Institutional performance	0.082	1.085	0.093	1.097
	(0.061)	(0.96–1.22)	(0.058)	(0.98–1.23)
Trust in scientists	0.818^***^	2.265^***^	0.856^***^	2.355^***^
	(0.048)	(2.06–2.49)	(0.048)	(2.14–2.59)
Scientific literacy	−0.018	0.982	−0.034	0.966
	(0.036)	(0.92–1.05)	(0.034)	(0.90–1.03)
Medical expert	0.214^**^	1.238^**^	0.182^**^	1.200^**^
	(0.063)	(1.10–1.40)	(0.059)	(1.07–1.35)
Traditional media	−0.034	0.967	−0.042	0.959
	(0.063)	(0.85–1.09)	(0.059)	(0.85–1.08)
Biomedical journal	−0.049	0.952	0.004	1.004
	(0.062)	(0.84–1.07)	(0.058)	(0.90–1.12)
Social media	0.059	1.061	0.049	1.050
	(0.059)	(0.94–1.19)	(0.055)	(0.94–1.17)
**Control variable**				
Perceived likelihood	−0.020	0.980	−0.013	0.987
	(0.047)	(0.89–1.07)	(0.044)	(0.91–1.08)
Perceived severity	0.122^**^	1.130^**^	0.109^**^	1.115^**^
	(0.042)	(1.04–1.23)	(0.040)	(1.03–1.21)
Age	0.034	1.034	−0.009	0.991
	(0.058)	(0.92–1.16)	(−0.55)	(0.89–1.10)
Gender	−0.287^*^	0.750^*^	−0.379^***^	0.684^***^
	(0.113)	(0.60–0.94)	(0.106)	(0.56–0.84)
Monthly income	0.086	1.090	0.089	1.093
	(0.071)	(0.95–1.25)	(0.066)	(0.96–1.24)
Education	0.101	1.107	0.042	1.043
	(0.059)	(0.99–1.24)	(0.056)	(0.93–1.16)
Log likelihood	−1227.518		−1368.343	
LR Chi^2^	454.70(0.000)		505.13(0.000)	
Nagelkerke *R*^2^	0.263		0.276	
Classification accuracy	82.7%		82.6%	
*N*	2,038		2,038	

Adjusted OR (95% CI) = adjusted odds ratio and the corresponding 95% confidence interval.

^*^p < 0.05, ^**^p < 0.01, ^***^p < 0.001.

**Table 3:** Regression results of attitudes toward US vaccines.

	**Safety (US)**		**Effectiveness (US)**	
	**(Model 3)**		**(Model 4)**	
	β_3_ **(SE)**	**Adjusted OR**_3_ **(95%CI)**	β_4_ **(SE)**	**Adjusted OR**_4_ **(95%CI)**
**Key variables**				
Institutional performance	−0.070	0.932	−0.093^*^	0.911^*^
	(0.047)	(0.85–1.02)	(0.048)	(0.83–1.00)
Trust in scientists	−0.082^*^	0.921^*^	−0.123^***^	0.884^***^
	(0.035)	(0.86–0.99)	(0.035)	(0.82–0.95)
Scientific literacy	−0.047	0.954	−0.039	0.962
	(0.027)	(0.91–1.01)	(−0.027)	(0.91–1.01)
Medical expert	−0.108^*^	0.898^*^	−0.099^*^	0.906^*^
	(0.046)	(0.82–0.98)	(0.046)	(0.83–0.99)
Traditional media	−0.075	0.927	−0.068	0.934
	(0.046)	(0.85–1.02)	(0.047)	(0.85–1.02)
Biomedical journal	0.107^*^	1.112^*^	0.093^*^	1.098^*^
	(0.046)	(1.02–1.22)	(0.046)	(1.00–1.20)
Social media	0.065	1.067	0.073	1.076
	(0.042)	(0.98–1.16)	(0.043)	(0.99–1.17)
**Control variable**				
Perceived likelihood	0.420^***^	1.521^***^	0.421^***^	1.522^***^
	(0.036)	(1.42–1.63)	(0.036)	(1.42–1.63)
Perceived severity	−0.037	0.964	−0.021	0.979
	(0.032)	(0.90–1.03)	(0.032)	(0.92–1.04)
Age	0.032	1.033	0.028	1.028
	(0.043)	(0.95–1.12)	(0.44)	(0.94–1.12)
Gender	−0.245^**^	0.783^**^	−0.301^***^	0.740^***^
	(0.084)	(0.66–0.92)	(0.084)	(0.63–0.87)
Monthly income	−0.026	0.974	0.006	0.994
	(0.052)	(0.88–1.08)	(0.052)	(0.90–1.10)
Education	0.077	1.080	0.082	1.086
	(0.044)	(0.99–1.18)	(0.044)	(0.99–1.18)
Log likelihood	−2707.862		−2698.923	
LR Chi^2^ (*p*–value)	201.42(0.000)		223.33(0.000)	
Nagelkerke *R*^2^	0.100		0.111	
Classification accuracy	82.7%		82.6%	
*N*	2,038		2,038	

Adjusted OR (95% CI) = adjusted odds ratio and the corresponding 95% confidence interval.

^*^p < 0.05, ^**^p < 0.01, ^***^p < 0.001.

Our findings reveal that respondents' evaluation of government performance in dealing with the epidemic has a positive influence on the perceived safety and effectiveness of domestic vaccines (adjusted odds ratio (aOR)_1_ = 1.085, 95%CI: 0.96–1.22, P = 0.182; aOR_2_ = 1.097, 95%CI: 0.98–1.23, *P* = 0.109), while it is negatively correlated with confidence in US vaccines, and the correlation is significant in the perceived effectiveness of vaccines (aOR_3_ = 0.932, 95%CI: 0.85–1.02, *P* = 0.136; aOR_4_ = 0.911, 95%CI: 0.83–1.00, *P* = 0.050). Similarly, trust in scientists has a positive and significant effect on attitudes toward the safety and effectiveness of domestic vaccines (aOR_1_ = 2.265, 95%CI: 2.06–2.49, *P* < 0.000; aOR_2_ = 2.355, 95%CI: 0.14–2.59, *P* < 0.000), and leads to lower confidence in the US vaccines than Chinese ones (aOR_3_ = 0.921, 95%CI: 0.86–0.99, *P* = 0.019; aOR_4_ = 0.884, 95%CI: 0.82–0.95, *P* < 0.000).

The regression results of Models 1–4 reveal that scientific literacy has no significant effect on respondents' attitudes toward domestic and US vaccines (aOR_1_ = 0.982, 95%CI: 0.92–1.05, P = 0.608; aOR_2_ = 0.966, 95%CI: 0.90–1.03, *P* = 0.310; aOR_3_ = 0.954, 95%CI: 0.91–1.01, *P* = 0.077, aOR_4_ = 0.962, 95%CI: 0.91–1.01, *P* = 0.152). The results indicate that individuals' knowledge of vaccines, viruses, and biology has limited influence on their attitude toward COVID-19 vaccines of different origins.

The different sources of health information have different impacts on vaccine attitudes. As shown in Models 1–4, acquiring health information from medical experts more frequently will lead to higher confidence in the domestic vaccines than in those produced in the US (aOR_1_ = 1.238, 95%CI: 1.10–1.40, *P* < 0.01; aOR_2_ = 1.200, 95%CI: 1.07–1.35, *P* < 0.01; aOR_3_ = 0.898, 95%CI: 0.82–0.98, *P* < 0.05; aOR_4_ = 0.906, 95%CI: 0.83–0.99, *P* < 0.05). Neither the effects of traditional nor social media usage are so significant. However, the public's attitude toward US vaccines is significantly influenced by reading of biomedical journal (aOR_3_ = 1.112, 95%CI: 1.02–1.22, *P* < 0.05; aOR_4_ = 1.098, 95%CI: 1.00–1.20, *P* < 0.05). If someone obtains health information from medical periodicals more frequently, he/she will hold a more positive attitude toward the US vaccines when comparing them with Chinese vaccines. However, the influence of biomedical journals on the public's opinion of domestic vaccines is insignificant (aOR_1_ = 0.952, 95%CI: 0.84–1.07, *P* = 0.422; aOR_2_ = 1.004, 95%CI: 0.90–1.12, *P* = 0.951).

As for control variables, respondents perceived that likelihood of infection has no significant effect on the public's opinion of the domestic vaccines, but it leads to a higher confidence in US vaccines (see Models 1–4). Respondents with higher perception of the COVID-19 virus severity tend to manifestly trust in domestic vaccines, while this factor has no significant influence on their attitude toward US vaccines. Compared to male participants, females seem to be more hesitant about the safety and effectiveness of COVID-19 vaccines both from China and the US: the coefficients of gender are stably negative and significant in Models 1–4 (aOR_1_ = 0.750, 95%CI: 0.60–0.94, *P* < 0.05; aOR_2_ = 0.684, 95%CI: 0.56–0.84, *P* < 0.001; aOR_3_ = 0.783, 95%CI: 0.66–0.92, *P* < 0.01; aOR_4_ = 0.740, 95%CI: 0.63–0.87, *P* < 0.001). Respondents' age, monthly income, and education have no significant effects on their attitudes toward vaccines.

#### 3.2.2. Influencing factors and vaccination preference

Then we examined how institutional trust, scientific literacy, and information usage influence respondents' vaccination preferences for either vaccines originating in China or those from the US. The results are shown in Table 4. The values of the likelihood ratio chi-square of the Models 5 and 6 are 365.75 and 247.61, which are both statistically significant, as compared to null models. The classification accuracy (measured by the $R_{\text{count}}^{2}$ coefficient) of the two models is 82.6 and 64.9% respectively, indicating that the two models both have a good fit. The Nagelkerke *R*^2^ showed that, the Models 5 can explain 21.8% of the variation in the preference for Chinese vaccines, Models 6 explains 13.4% of the variation in the preference for the US vaccines.

**Table 4:** Regression results of vaccination preference.

	**Priority of vaccines**			
	**CN vaccines (Model 5)**		**US vaccines (Model 6)**	
	β_5_ **(SE)**	**Adjusted OR**_5_ **(95%CI)**	β_6_ **(SE)**	**Adjusted OR**_6_ **(95%CI)**
**Key variables**				
Institutional performance	0.219^***^	1.294^***^	−0.134^**^	0.874^**^
	(0.061)	(1.10–1.40)	(0.052)	(0.79–0.97)
Trust in scientists	0.583^***^	1.791^***^	−0.228^***^	0.796^***^
	(0.044)	(1.64–1.95)	(0.040)	(0.74–0.86)
Scientific literacy	−0.009	0.991	−0.058	0.944
	(0.039)	(0.92–1.07)	(0.030)	(0.89–1.00)
Medical expert	0.085	1.088	−0.080	0.923
	(0.069)	(0.95–1.25)	(0.051)	(0.84–1.02)
Traditional media	0.020	1.020	−0.120^*^	0.887^*^
	(0.71)	(0.89–1.17)	(0.052)	(0.80–0.98)
Biomedical journal	−0.141^*^	0.869^*^	0.104^*^	1.110^*^
	(0.069)	(0.76–0.99)	(0.051)	(1.00–1.23)
Social media	0.035	1.036	0.128^**^	1.137^**^
	(0.066)	(0.91–1.18)	(0.048)	(1.04–1.25)
**Control variable**				
Perceived likelihood	−0.231^***^	0.794^***^	0.419^***^	1.521^***^
	(0.051)	(0.72–0.88)	(0.037)	(1.41–1.63)
Perceived severity	0.206^***^	1.229^***^	−0.079^*^	0.924^*^
	(0.049)	(1.12–1.35)	(0.036)	(0.86–0.99)
Age	−0.098	0.907	0.056	1.057
	(0.065)	(0.80–1.03)	(0.048)	(0.96–1.16)
Gender	−0.227	0.797	−0.165	0.848
	(0.126)	(0.623–1.02)	(0.094)	(0.71–1.02)
Monthly income	0.031	1.031	−0.002	0.998
	(0.078)	(0.89–1.20)	(0.058)	(0.89–1.12)
Education	0.023	1.024	0.065	1.067
	(0.065)	(0.90–1.16)	(0.049)	(0.97–1.18)
Log likelihood	−1176.954		−1822.007	
Nagelkerke *R*^2^	0.218		0.134	
LR Chi^2^	365.75(0.000)		247.61 (0.000)	
Classification accuracy	82.6%		64.9%	
*N*	2,038		2,038	

Adjusted OR (95% CI) = adjusted odds ratio and the corresponding 95% confidence interval.

^*^p < 0.05, ^**^p < 0.01, ^***^p < 0.001.

A positive evaluation of Chinese government performance significantly influenced individuals' preference for taking domestic vaccines while reducing their willingness to take American vaccines (aOR_5_ = 1.294, 95%CI: 1.10–1.40, *P* < 0.001; aOR_6_ = 0.874, 95%CI: 0.79–0.97, *P* < 0.01). Similarly, respondents' trust in Chinese scientists could positively affect their preference to receive vaccines from China and significantly reduce their willingness to receive the US vaccines (aOR_5_ = 1.791, 95%CI: 1.64–1.95, *P* < 0.001; aOR_6_ = 0.796, 95%CI: 0.74–0.86, *P* < 0.001). In Models 5 and 6, the regression coefficients of scientific literacy on respondents are both negative but not significant, indicating that personal scientific literacy has a limited influence on vaccination preference (aOR_5_ = 0.991, 95%CI: 0.92–1.07, *P* = 0.813; aOR_6_ = 0.944, 95%CI: 0.89–1.00, *P* = 0.0541). Respondents' sources of information are important influencing factors for individuals' vaccination choice. In this regard, respondents' reliance on information provided by medical personnel, though not significant, has a positive influence on their preference for domestic vaccines and a negative influence on their preference for the US vaccines (aOR_5_ = 1.088, 95%CI: 0.95–1.25, *P* = 0.219; aOR_6_ = 0.923, 95%CI: 0.84–1.02, *P* = 0.117). Meanwhile, the usage of traditional media is significantly and negatively associated with the preference for US vaccines (aOR_6_ = 0.887, 95%CI: 0.80–0.98, *P* < 0.05), while the usage of social media is positively correlated with individuals' willingness to take US vaccines (aOR_6_ = 1.137, 95%CI: 1.04–1.25, *P* < 0.01). Frequency of acquiring information from biomedical journals is positively associated with preference for US vaccines and negatively affects acceptance of domestic vaccines (aOR_5_ = 0.869, 95%CI: 0.76–0.99, *P* < 0.05; aOR_6_ = 1.110, 95%CI: 1.00–1.23, *P* < 0.05).

As for risk perceptions, respondents' perceived likelihood of infection has a negative effect on their preference for domestic vaccines but a positive effect on their preference for US vaccines (aOR_5_ = 0.794, 95%CI: 0.72–0.88, *P* < 0.001; aOR_6_ = 1.521, 95%CI: 1.41–1.63, *P* < 0.001). On the other hand, perceived severity motivates individuals' willingness to receive domestic vaccines and lowers their prioritization of taking US vaccines. The demographic variables of age, gender, monthly income, and education degree all have no significant influence on public attitudes toward vaccinating with either Chinese or US vaccines.

The results in Table 3 accord with those in Table 2 in showing that the effects of trust in scientists are still significant, motivating respondents to take shots at COVID-19 vaccines produced domestically (see Models 5–6). And accessing health information from biomedical journals promotes respondents' preference for US vaccines. In addition, the effect of individuals' subjective evaluation of government performance becomes significant in relation to vaccination preference, leading respondents to prefer domestic vaccines to US ones. Furthermore, the influence of social media and media both have significant effects on individuals' preference for US vaccines. Usage of traditional media tends to discourage people from getting US vaccines, while usage of social media increases willingness to do so.

#### 3.2.3. The relationship between perception of vaccine quality and vaccination preference

To examine whether individuals' vaccination preferences are related to their evaluation of different vaccines, we added the comparison of the perceived safety and effectiveness between the US and Chinese vaccines in the model, respectively. And the results are displayed in Tables 5, 6. The LR test showed that the Models 7–10 are all statistically significant, as compared to the null models. The classification accuracy of the four models reaches 82.7, 82.6, 76.4, and 77.0% respectively, indicating that the four models have a good fit. After added the comparison variables, the Models 9 and 10 can explain 59.0 and 61.1% of the variation in the preference for the US vaccines, which has increased greatly when compared with Model 6. As the regression outcome showed, the participants' judgements of different vaccines exert significant influence on the prioritization of US vaccines (see Models 9–10), but have no impact on their preference for domestic vaccines (see Models 7–8). Meanwhile, trust in government and scientists both significantly promote a preference for domestic vaccines and decrease individuals' willingness to select US vaccines (see Models 7–10). This further indicates that the Chinese public's recognition of the domestic vaccines is not affected by the vaccine quality itself, but from their trust in the domestic institutions.

**Table 5:** The effect of perceived quality on vaccination preference for domestic vaccines.

	**Vaccination preference (CN)**		**Vaccination preference (CN)**	
	**(Model 7)**		**(Model 8)**	
**Key variables**	β_7_ **(SE)**	**Adjusted OR**_7_ **(95%CI)**	β_8_ **(SE)**	**Adjusted OR**_8_ **(95%CI)**
Safety comparison	−0.044	0.957		
	(0.051)	(0.87–1.06)		
Effectiveness comparison			−0.002	0.998
			(0.051)	(0.90–1.10)
Institutional performance	0.218^***^	1.243^***^	0.219^***^	1.245^***^
	(0.061)	(1.10–1.40)	(0.061)	(1.10–1.40)
Trust in scientists	0.582^***^	1.790^***^	0.583^***^	1.791
	(0.044)	(1.64–1.95)	(0.044)	(1.64–1.95)
Scientific literacy	−0.011	0.989	−0.009	0.991
	(0.039)	(0.92–1.07)	(0.039)	(0.92–1.07)
Medical expert	0.082	1.086	0.085	1.088
	(0.069)	(0.95–1.24)	(0.069)	(0.95–1.25)
Traditional media	0.017	1.017	0.020	1.020
	(0.071)	(0.89–1.17)	(0.071)	(0.89–1.17)
Biomedical journal	−0.137^*^	0.872^*^	−0.141^*^	0.869^*^
	(0.069)	(0.76–1.00)	(0.069)	(0.76–1.00)
Social media	0.038	1.039	0.036	1.036
	(0.066)	(0.91–1.18)	(0.066)	(0.91–1.18)
Control variable	Controlled		Controlled	
Log likelihood	−1176.583		−1176.953	
LR Chi^2^	357.49 (0.000)		356.75 (0.000)	
Nagelkerke *R*^2^	0.219		0.218	
Classification accuracy	82.7%		82.6%	
*N*	2,038		2,038	

Adjusted OR (95% CI) = adjusted odds ratio and the corresponding 95% confidence interval.

^*^p < 0.05, ^***^p < 0.001.

**Table 6:** The effect of perceived quality on vaccination preference of US vaccines.

	**Vaccination preference (US)**		**Vaccination preference (US)**	
	**(Model 9)**		**(Model 10)**	
**Key variables**	β_9_ **(SE)**	**Adjusted OR**_9_ **(95%CI)**	β_10_ **(SE)**	**Adjusted OR**_10_ **(95%CI)**
Safety comparison	1.647^***^	5.192		
	(0.063)	(4.59–5.86)		
Effectiveness comparison			1.742^***^	5.708^***^
			(0.065)	(5.03–6.48)
Institutional performance	−0.161^*^	0.851^*^	−0.132^*^	0.876^*^
	(0.063)	(0.75–0.96)	(0.065)	(0.77–0.99)
Trust in scientists	−0.277^***^	0.758^***^	−0.232^***^	0.793^***^
	(0.046)	(0.69–0.83)	(0.046)	(0.72–0.87)
Scientific literacy	−0.026	0.974	−0.035	0.966
	(0.037)	(0.91–1.05)	(0.037)	(0.90–1.04)
Medical expert	−0.040	0.960	−0.064	0.938
	(0.064)	(0.85–1.09)	(0.065)	(0.83–1.07)
Traditional media	−0.098	0.907	−0.090	0.914
	(0.064)	(0.80–1.03)	(0.065)	(0.80–1.04)
Biomedical journal	0.070	1.073	0.068	1.070
	(0.064)	(0.95–1.22)	(0.065)	(0.94–1.22)
Social media	0.117^*^	1.125^*^	0.092	1.096
	(0.059)	(1.00–1.26)	(0.060)	(0.97–1.23)
Control variable	Controlled		Controlled	
Log likelihood	−1233.827		−1196.376	
LR Chi^2^	1423.97		1498.88	
Nagelkerke *R*^2^	0.590		0.611	
Classification accuracy	76.4%		77.0%	
*N*	2,038		2,038	

Adjusted OR (95% CI) = adjusted odds ratio and the corresponding 95% confidence interval.

^*^p < 0.05, ^***^p < 0.001.

## 4. Discussion

This study shed lights on differences in Chinese attitudes toward COVID-19 vaccines from China and the United States by examining the issue in the context of an emergency situation. Analysis of the results revealed that, compared to American COVID-19 vaccines, most respondents have greater trust in Chinese COVID-19 vaccines, whether in terms of perceived effectiveness or safety, and they are more willing to take domestic vaccines. These findings are not only in contrast with the scientific facts (scientific studies have shown that the efficacy of Chinese COVID-19 vaccines such as Sinovac and Sinopharm is around 86%, compared to 95% for Pfizer and 94% for Moderna) (13, 14), but also with previous published data. For example, based on a meta-analysis of 58 articles about Chinese public attitudes toward various vaccines, Wang et al. (10) found that almost one half of respondents don't trust domestic vaccines' effectiveness, nor their safety, and that they were more willing to take imported vaccines where these were available. Another study, about Chinese parents' and caregivers' attitudes toward foreign and domestic vaccines for children, showed that respondents found the two comparable and as having similar levels of effectiveness and safety (11).

The contrasting findings may be explained by the context in which the studies were conducted: previous studies evaluated vaccine attitudes during normal periods when individuals had access to a wealth of evidence-based information to make objective choices. However, during emergency situations, access to consistent and evidence-based information is limited, and individuals' attitudes toward unknown vaccines may be more influenced by psychological factors such as socio-political beliefs (15, 17). This study empirically examines the role of knowledge, information channels, and socio-political beliefs in shaping COVID-19 vaccine attitudes during an emergency situation. The results of our analysis show that there was no significant correlation between scientific literacy and respondents' attitudes toward domestic and American COVID-19 vaccines. On the other hand, it was shown that institutional trust strongly affects respondents' attitudes toward the two types of vaccines, and that the influence of information sources changes in nuanced ways according to the type of the channel that an individual frequents.

### 4.1. The limited influence of scientific literacy

In this study, we did not find a stable and significant correlation between scientific literacy and respondents' attitudes toward domestic and American COVID-19 vaccines. Strong disagreement with the statement “The quality of US vaccines is superior to that of domestic vaccines” is widespread, and there are no significant differences among groups according to age or income.

Possible explanations for these results are as follows. Firstly, due to the high levels of complexity involved in the production of COVID-19 vaccines, the sort of everyday biomedical knowledge possessed by most respondents is insufficient to evaluate objectively a previously unknown vaccine. Secondly, existing studies indicate that individual vaccine attitudes are complex and context-specific, and are influenced by a variety of psychological and socio-political factors beyond knowledge and information. For example, based on a systematic literature review of 38 article across 15 countries, Yaqub et al. (17) found that many of the reasons reported for vaccine attitudes are not related to ignorance or lack of awareness. Instead, their literature review found distrust of public health authorities (i.e., doctors, experts/researchers, government officials) as a key determinant of vaccine hesitancy or distrust (12). Other studies found that political ideology also directly influences vaccine attitudes. For example, people who are politically conservatives are more likely to be vaccine skeptics than other individuals (15).

In this regard, our findings around the non-significant influence of scientific literacy on people's vaccine attitudes are in accordance with previous studies. They support previous findings that the influence of scientific literacy is limited by institutional trust and the sources from which people receive information about vaccine risks and benefits.

### 4.2. The central role of institutional trust

The COVID-19 vaccines are totally new and had been developed in a very short time. People lack sufficient and reliable evidence-based data about their effectiveness and safety, especially in relation to side effects, to make a rational choice, in contrast with the high levels of information available about other vaccines, such as those for measles-mumps-rubella or polio, diseases with which people are more familiar. Furthermore, countries such as the US and European countries have published information about the side effects of COVID-19 vaccines, and Chinese media have reported a large quantity of stories about the thrombus, Bell's palsy, and deaths caused by these vaccines, especially the Pfizer vaccine in the spring of 2021 (37). Meanwhile, the Chinese Center for Disease Control and Prevention only published data about the safety and side effects of domestic COVID-19 vaccines on 28 May 2021, and this was the only occasion on which such information was published in 2021 (38). Such imbalanced information delivery may have disrupted and confused many people's perceptions of the quality and safety of domestic and foreign vaccines. Previous knowledge and experiences in this regard could no longer serve as references for the lay public to assess the COVID-19 vaccines.

Several authors have pointed out that when conflicting ‘scientific facts' exist and individuals need to choose between them, lay people turn to authorities who are considered experts in the relevant field to find answers (39). In other words, trust in scientific institutions and public authorities plays a central role in shaping individuals' perceptions of vaccines and intentions to be vaccinated.

In our study, the statistical results in Tables 2, 3, as well as the marginal effects (see Appendix) show that institutional trust plays a significant role in people's perceptions of vaccines. It is suggested that the more they trust in Chinese medical experts/scientists, the more confidence they have in the safety and effectiveness of domestic COVID-19 vaccines, the more willing they are to take domestic vaccines, and the less likely they are to prefer American vaccines (see Tables 3, 4). Furthermore, individuals' vaccination preferences are also significantly influenced by their evaluation of government performance. People with positive evaluations of the government's performance in controlling the spread of the pandemic prefer to get vaccinated with domestic vaccines and deprioritize the usage of American vaccines.

The regression results indicated that the more people trust in a specific government or scientific institutions, the more likely they are to demonstrate support for the vaccines from that country. Precisely speaking, the remarkable achievements that China had made in controlling the spread of the pandemic until May 2021, as well as the country's rapid economic development in recent decades, has encouraged a high level of trust in the government among Chinese people. In addition, public demonstrations of vaccination by medical experts and government leaders may also have boosted public confidence in domestic COVID-19 vaccines. On the other hand, the uncontrolled spread of COVID-19 and the high infection and mortality rates in the United States, as well as the deterioration in Chinese-US relations in recent years, are all likely to have reduced Chinese public trust in the capabilities of the US government, with this in turn affecting trust in American vaccines.

### 4.3. The selective influence of information sources

There exist a variety of information sources about Chinese and US vaccines, with each shaping attitudes in different ways and producing different levels of public trust in different vaccines (29, 40). People visit information sources they trust, and vaccine attitudes are shaped accordingly. For example, the higher the frequency with which people receive health-related information from doctors, the greater their perceptions of the relative safety and effectiveness of Chinese compared to American vaccines, and the higher respondents' trust in domestic vaccines. Our study results also show that use of traditional information sources is negatively related to their preference for US vaccines. When facing severe public crisis, the Chinese local authorities are accustomed to developed various strategies to positively shape public opinions (41). In the epidemic, doctors and traditional mass media are officially institutional agents, they are in charge of missions to transmit to members of the public the governments' policies and discourses. So, trust in doctors and traditional media can be regarded as an extension of institutional trust, and therefore as helping to shape publics attitudes toward different vaccines.

Social media has played the opposite role. In our study, the more likely people were to use social media to acquire health-related information, the more likely they were to demonstrate support for American COVID-19 vaccines. Social media is well-known for its rebellious approach to public information communication. Previous studies have pointed out that social media has facilitated the spread of misinformation about vaccines (42), which generalizes to vaccine distrust and vaccination hesitancy (43, 44). The results of our study present a nuanced picture of the influence of social media on people's vaccine attitudes: rather than being a disruptor to vaccine trust-building, social media serves as a checking and balancing platform. Its influence on public attitudes toward vaccines might depend on the specific institutional context of a society. In a country such as China, where the government dominate communication channels, and traditional information sources are under strict regulation, social media provides citizens a relatively open and free space for acquiring information different from that communicated by the government and health agencies (28). This kind of information is not necessarily false or distorted; it is simply different from that provided by the government. More exposure to social media might lead audiences to access information from different sources, besides government propaganda messages, such as information reported by foreign media and online anti-government content. Information about vaccines does not speak for itself; the type of information source through which it is communicated shapes how it is interpreted and used (22). Therefore, absorbing information from multiple sources makes it more unlikely that government propaganda will dominate public opinion and helps to reduce bias around vaccines. The regression results verified that use of social media significantly increases respondents' willingness to accept the US vaccines.

The third type of information sources assessed by our research is biomedical journals. the results of our analysis show that reading biomedical journals is negatively correlated with prioritization of domestic COVID-19 vaccines, and positively correlated with more positive attitudes toward American vaccines. This finding is not surprising. Those who seek to obtain reliable information about COVID-19 vaccines through professional journals are likely to possess a certain level of biomedical knowledges and a capacity for understanding related professional language. In addition, scientific articles published in biomedical journals provide scientific information that reflects in most cases the objective facts. Access to scientific journals constitutes one of the most effective resolutions to solving the information/knowledge-deficit dilemma regarding vaccine mistrust. However, their audience is limited due to the relatively high threshold in medical literacy required to read them.

These findings show that the influence of information sources on people's vaccine attitudes is selective and is positively related to the level of an individual's trust in the information source in question. People tend to trust selectively and to interpret information in ways that reflect personal political beliefs or degree of scientific literacy, and vaccine attitudes are also shaped in this way.

## 5. Strengths and limitations of the study

People's vaccine attitudes are context-specific, and factors that shape attitudes toward COVID-19 vaccines have been extensively investigated worldwide across various societal and cultural contexts. Some studies in developing countries have delved further into the extent to which factors such as price and brand can influence vaccination preferences (8, 45, 46). However, few studies have examined vaccine attitudes during an emergency, a unique situation that can shape psychological behaviors. This research sheds light on differences and potential explanatory factors in Chinese attitudes toward COVID-19 vaccines from China and the United States during the global outbreak period in spring 2021. It adds to the literature on the complex and dynamic attitudes toward different vaccines in specific situations, and raises awareness among policymakers about the significant influence of emotions and socio-political beliefs on vaccination decisions during an emergency.

It is important to note that this study was conducted in a specific political context where vaccine administration and communication is dominated by the state. The information scarcity caused by the emergency situation certainly contributes to the public's psychological reliance on public institutions. However, it should also be acknowledged that Chinese vaccine attitudes have also been shaped by a lack of transparent information communication and limited vaccination options (imported COVID-19 vaccines were not available to the general public in China), which may have limited individuals' ability to make objective choices. Given the specific political characteristics of the study area, it remains to be seen whether these findings are applicable to other countries outside of authoritarian regimes such as mainland China and further research is needed to investigate this.

## 6. Conclusion

This study has investigated differences in the attitudes of Chinese residents toward COVID-19 vaccines produced in China and the United States, as well as possible explanations for these differences, by examining them in the context of an emergency situation. In contrast with the findings of previous studies, which have shown that Chinese people trust more or at least equally imported vaccines compared to domestic ones, this research shows that in a situation in which reliable information on which to base decision making is scarce and insufficient and people need to make key decisions to deal with the threat of an immediate and substantial risk, most Chinese residents trust more in the safety and effectiveness of domestic vaccines than US ones. This trust gap does not result from disparity in the actual quality and safety of the different vaccines *per se*, but is instead closely bound up with people's trust in domestic institutions. Rather than relying on objective information and knowledge, such as that based on scientific literacy, Chinese attitudes toward the origins of different vaccines are, in an emergency context, more influenced by socio-political beliefs.

## Data availability statement

The raw data supporting the conclusions of this article will be made available by the authors, without undue reservation.

## Ethics statement

The studies involving human participants were reviewed and approved by Academic Ethics Committee of Zhejiang Gongshang University. The Ethics Committee waived the requirement of written informed consent for participation.

## Author contributions

YY, ZS, and CS: conceptualization, data curation, supervision, and validation. YY and CS: formal analysis, methodology, original draft, and writing—review and editing. All authors contributed to the article and approved the submitted version.

## References

[1] Walach H Klement RJ Aukema W. safety of COVID-19 Vaccinations—We should rethink the policy. Vaccines. (2021) 9:693. 10.3390./vaccines9070693 34202529 PMC8294615

[2] Larson HJ Clarke RM Jarrett C. Measuring trust in vaccination: a systematic review. Human Vacc Immunotherap. (2018) 14:1599–609. 10.1080/21645515.2018.1459252 29617183 PMC6067893

[3] Dubé E Gagnon D MacDonald N. Underlying factors impacting vaccine hesitancy in high income countries: a review of qualitative studies. Expert Rev Vacc. (2018) 3:1406. 10.1080./14760584.2018.1541406 30359151

[4] El-Far Cardo A Kraus T Kaifie A. Factors that shape people's attitudes towards the COVID-19 pandemic in Germany—The influence of MEDIA politics and personal characteristics. Int J Environ Res Public Health. (2021) 18:7772. 10.3390/ijerph18157 34360063 PMC8345618

[5] Jamieson H Romer D Jamieson PE Winneg KM Pasek J. The role of non–covid-specific and covid-specific factors in predicting a shift in willingness to vaccinate: a panel study. Proceed Nat Acad Sci. (2021) 118:6118. 10.1073./pnas.2112266118 34930844 PMC8719857

[6] Elnaem MH Mohd Taufek NH Ab Rahman NS Mohd Nazar NI Zin CS Nuffer W. COVID-19 vaccination attitudes perceptions and side effect experiences in malaysia: do age gender and vaccine type matter? Vaccines. (2021) 9:1156. 10.3390/vaccines9101156 34696264 PMC8539146

[7] Park HK Ham JH Jang DH Lee JY Jang WM. Political Ideologies government trust and COVID-19 vaccine hesitancy in South Korea: a cross-sectional survey. Int J Environ Res Public Health. (2021) 18:10655. 10.3390/ijerph182010655 34682401 PMC8536119

[8] Rzymski P Zeyland J Poniedziałek B Małecka I Wysocki J. The perception and attitudes toward COVID-19 vaccines: a cross-sectional study in Poland. Vaccines. (2021) 9:382. 10.3390/vaccines9040382 33919672 PMC8069794

[9] Wijerathne HDHP Purijjala IWCD Pathirana DSA Kumarasena KKS. Exploring COVID-19 vaccine hesitancy and its antecedents: a descriptive study of young adults in Sri Lanka. Conference Paper Oct. (2022). Available online at: https://www.researchgate.net/publication/366482688 (accessed January 11, 2023).

[10] Wang X Lu Q Hou Z. Vaccine confidence and vaccination attitude and willingness among Chinese residents: a systematic review. Chin J Public Health. (2020) 35:6270. 10.11847./zgggws1126270

[11] Huang Z Sun X Wagner AL. Parent and caregiver perceptions about the safety and effectiveness of foreign and domestic vaccines in Shanghai China. PLoS ONE. (2018) 13:e0197437. 10.1371/journal.pone.0197437 29782508 PMC5962069

[12] Al-Qerem WA Jarab AS. COVID-19 vaccination acceptance and its associated factors among a middle eastern population. Front Public Health. (2021) 9:632914. 10.3389/fpubh.2021.632914 33643995 PMC7902782

[13] Our World in Data. Coronavirus (COVID-19) Vaccinations-Statistics and Research 2021. Available online at: https://ourworldindata.org/covid-vaccinations (accessed August 10, 2021).

[14] Alqassieh R Suleiman A Abu-Halaweh S. Pfizer-BioNTech and sinopharm: a comparative study on post-vaccination antibody titers. Vaccines. (2021) 9:1223. 10.3390/vaccines9111223 34835153 PMC8620087

[15] Baumgaertner B Carlisle JE Justwan F. The influence of political ideology and trust on willingness to vaccinate. PLoS ONE. (2018) 13:e0191728. 10.1371/journal.pone.0191728 29370265 PMC5784985

[16] Motoki K Saito T Takano Y. Scientific literacy linked to attitudes toward COVID-19 vaccinations: a pre-registered study. Front. Commun. (2021) 6:707391. 10.3389/fcomm.2021.707391

[17] Yaqub O Castle-Clarke S Sevdalis N Chataway J. Attitudes to vaccination: a critical review. Soc Sci Med. (2014) 112:18. 10.1016/j.socscimed.04018 24788111

[18] Mishra JL Allen DK Pearman AD. Understanding decision making during emergencies: a key contributor to resilience. EURO J Dec Proce. (2015) 3:397–424. 10.1007/s40070-015-0039-z

[19] Cvetkovich G Siegrist M Murray R Tragesser S. New information and social trust: asymmetry and perseverance of attributions about hazard managers. Risk Anal. (2002) 22:359–67. 10.1111/0272-4332.00030 12022682

[20] Woko C Siegel L Hornik R. An investigation of low COVID-19 vaccination intentions among Black Americans: the role of behavioral beliefs and trust in COVID-19. Inform Sou J Health Commun. (2020) 25:10.819–26. 10.1080/10820201864521 33719874

[21] Chanel O. Impact of information on intentions to vaccinate in a potential epidemic: swine-origin Influenza A (H1N1). Soc Sci Med. (2011) 72:142e−8. 10.1016/j.socscimed.2010.11.018 21163566

[22] Brown J Duguid P. The Social Life of Information. MA: Harvard Business Press Harvard (2002).

[23] Zeng Y. Home work homework and field. Antrhopol News June. (2020) 12:2020. Available online at: https://anthrodendum.org/2020/06/12/home-work-homework-and-fieldwork/ (accessed July 21, 2022).

[24] Sturgis P Brunton-Smith I Jackson J. Trust in science social consensus and vaccine confidence. Nat Hum Behav. (2021) 3:1–7. 10.1038./s41562-021-01115-7 34002053

[25] Azlan AA Hamzah MR Sern TJ Ayub SH Mohamad E. Public knowledge attitudes and practices towards COVID-19: a cross-sectional study in Malaysia. PLoS ONE. (2020) 15:e0233668. 10.1371/journal.pone.0233668 32437434 PMC7241824

[26] Fotou N Constantinou M. The role of health and biology literacy in the era of the COVID-19 pandemic. ASE Int. (2020) 11:29–33. 10.1590/1806-9282.66.S2.31 32965352

[27] Lueck JA Callaghan T. Inside the 'black box' of covid-19 vaccination beliefs: revealing the relative importance of public confidence and news consumption habits. Soc Sci Med. (2022) 298:114874. 10.1016/j.socscimed.2022.114874 35278975 PMC8885110

[28] Liu Z Janet Z Yang JZ. In the Wake of Scandals: How media use and social trust influence risk perception and vaccination intention among Chinese parents. Health Commun. (2020) 3:8834. 10.1080/10420201748834 32264705

[29] Catellier JRA Yang ZJ. Trust and affect: how do they impact risk information seeking in a health context?. J Risk Res. (2012) 15:1–15. 10.1080/13669877.2012.686048

[30] Furini M. Identifying the features of provax and novax groups from social media conversations. Comp Human Behav. (2021) 3:5. 10.1016./j.chb.2021.106751

[31] Mir HH Parveen S Mullick NH Nabi S. Using structural equation modeling to predict indian people's attitudes and intentions towards covid-19 vaccination. Diabetes and metabolic syndrome. Clin Rese Rev. (2021) 15:1017–22. 10.1016/j.dsx.2021.05.006 34000711 PMC8105307

[32] Dror A.A Eisenbach N Taiber S Morozov N. G Sela E. Vaccine hesitancy: the next challenge in the fight against covid-19. Eu J Epidemiol.(2020) 35:8. 10.1007/s10654-020-00671-y 32785815 PMC8851308

[33] Caserotti M Girardi P Rubaltelli E Tasso A Lotto L Gavaruzzi T. Associations of COVID-19 risk perception with vaccine hesitancy over time for Italian residents. Soc Sci Med. (2021) 272:113688. 10.1016/j.socscimed.2021.113688 33485215 PMC7788320

[34] Gehrau V Fujarski S Lorenz H Schieb C Blöbaum B. The impact of health information exposure and source credibility on COVID-19 vaccination intention in Germany. Int J Environ Res Public Health. (2021) 18:4678. 10.3390/ijerph18094678 33924796 PMC8124400

[35] Brian A O'Shea U eda M. Who is more likely to ignore experts' advice related to COVID-19? Prevent Med Rep. (2021) 23:101470. 10.1016/j.pmedr.2021.101470 34277330 PMC8261004

[36] Schwarzinger M Watson V Arwidson P Alla F Luchini S. COVID-19 vaccine hesitancy in a representative working-age population in France: a survey experiment based on vaccine characteristics. Lancet Public Health Apr;6. (2021) 3:e210–21. 10.1016/S2468-2667(21)00012-8 33556325 PMC7864787

[37] Yu G Yang Y Chen X. The perception willingness and influencing factors of vaccination among national residents in the platform perspective. J Mass Commun. (2021) 7:64–72. 10.15897/j.cnki.cn51-1046/g2.20210625.004

[38] Chinese Center for Disease Control and Prevention. Monitoring information about the adverse effects caused by the COVID-19 vaccination in China. Available online at: www.chinacdc.cn/jkzt/ymyjz/ymyjjz_6758/202105/t20210528_230911.html (accessed June 25, 2022).

[39] Taylor AH Rupali JL Jennifer EG. Development of a scale to measure trust in public health authorities: prevalence of trust and association with vaccination. J Health Commun. (2021) 26:272–80. 10.1080/10820211927259 33998402 PMC8225577

[40] Chryssochoidis G Strada A Krystallis A. Public trust in institutions and information sources regarding risk management and communication: Towards integrating extant knowledge. J Risk Res. (2009) 12:137–85. 10.1080/13669870802637000

[41] Repnikova. Information management during crisis events: a case study of Beijing floods of 2012. J Contemp China. (2017) 26:711–25. 10.1080/10670564.2017.1305503

[42] Chou W-YS Oh A Klein WMP. Addressing health-related misinformation on social media. JAMA. (2018) 320:2417–48. 10.1001/jama.2018.16865 30428002

[43] Schiavo R. Vaccine communication in the age of COVID-19: getting ready for an information war. J Commun Healthc. (2020) 13:73–5. 10.1080/17538068.2020.1778959

[44] Broniatowski DA Jamison AM Qi S AlKulaib L Chen T Benton A Dredze M. Weaponized health communication: Twitter bots and Russian trolls amplify the vaccine debate. Am J Public Health. (2018) 108:1378–84. 10.2105/AJPH.2018.304567 30138075 PMC6137759

[45] Sarwar A Nazar N Nazar N Qadir A. Measuring vaccination willingness in response to COVID-19 using a multi-criteria-decision making method. Human Vacc Immunotherap. (2021) 17:12 4865–72. 10.1080/21645515.2021.2004836 34856879 PMC8903990

[46] Kiran T Junaid K.P Sharma D Jain L Vij J Satapathy P. Sociodemographic determinants of willingness and extent to pay for COVID-19 vaccine in India. Front. Public Health. (2022) 10:870880. 10.3389/fpubh.2022.870880 35734756 PMC9207713

