# Supplementary material for: Institutional trust, scientific literacy, and information sources: What factors determine people's attitudes toward COVID-19 vaccines of different origins in China?
Source: Front Public Health. 2023 Feb 20;11:1092425. doi: 10.3389/fpubh.2023.1092425 (PMC9986272; doi:10.3389/fpubh.2023.1092425)
Supplement: Supplementary file 1 [file Table_1.pdf]

Table 1: The marginal effect of scientist trust (Model1-Model6)

	Scientist trust					
	Model1	Model2	Model3	Model4	Model5	Model6
Strongly disagree	-0.0027*** (0.0006)	-0.0028*** (0.0007)	0.0188* (0.0080)	0.0280*** (0.0080)	-0.0081*** (0.0012)	0.0481*** (0.0082)
Somewhat disagree	-0.0038*** (0.0010)	-0.0046*** (0.0012)	-0.0009* (0.0005)	-0.0019** (0.0007)	-0.0050*** (0.0010)	-0.0102*** (0.0019)
Neither agree or disagree	-0.0253*** (0.0026)	-0.0343*** (0.0040)	-0.0080* (0.0034)	-0.0112** (0.0032)	-0.0207*** (0.0022)	-0.0362*** (0.0062)
Somewhat agree	-0.0888*** (0.0051)	-0.0979*** (0.0054)	-0.0036* (0.0015)	-0.0059** (0.0017)	-0.0360*** (0.0032)	-0.0011** (0.0004)
Strongly agree	0.1206*** (0.0061)	0.1398*** (0.0065)	-0.0063* (0.0027)	-0.0089** (0.0026)	0.0697*** (0.0049)	-0.0006* (0.0003)
