# Supplementary material for: Institutional trust, scientific literacy, and information sources: What factors determine people's attitudes toward COVID-19 vaccines of different origins in China?
Source: Front Public Health. 2023 Feb 20;11:1092425. doi: 10.3389/fpubh.2023.1092425 (PMC9986272; doi:10.3389/fpubh.2023.1092425)
Supplement: Supplementary file 2 [file Table_2.pdf]

Table 2: The marginal effect of medical expert (Model1-Model4)

	Medical expert			
	Model1	Model2	Model3	Model4
Strongly disagree	-0.0007**<br>(0.0003)	-0.0006*<br>(0.0002)	0.0246*<br>(0.0103)	0.0225*<br>(0.0104)
Somewhat disagree	-0.0010*<br>(0.0004)	-0.0010*<br>(0.0004)	-0.0012*<br>(0.0006)	-0.0015*<br>(0.0008)
Neither agree or disagree	-0.0066**<br>(0.0020)	-0.0073**<br>(0.0024)	-0.0105*<br>(0.0044)	-0.0090*<br>(0.0042)
Somewhat agree	-0.0232**<br>(0.0068)	-0.0208**<br>(0.0066)	-0.0047*<br>(0.0020)	-0.0048*<br>(0.0022)
Strongly agree	0.0315**<br>(0.0092)	0.0297**<br>(0.0095)	-0.0082*<br>(0.0035)	-0.0072*<br>(0.0034)
