# Supplementary material for: Institutional trust, scientific literacy, and information sources: What factors determine people's attitudes toward COVID-19 vaccines of different origins in China?
Source: Front Public Health. 2023 Feb 20;11:1092425. doi: 10.3389/fpubh.2023.1092425 (PMC9986272; doi:10.3389/fpubh.2023.1092425)
Supplement: Supplementary file 3 [file Table_3.pdf]

Table 3: The marginal effect of institutional trust (Model7-Model10)

| | Institutional performance | | | |
|---|---|---|---|---|
| | Model7 | Model8 | Model9 | Model10 |
| Strongly disagree | -0.0023**<br>(0.0009) | -0.0025**<br>(0.0009) | 0.0186*<br>(0.0073) | 0.0147*<br>(0.007) |
| Somewhat disagree | -0.0014*<br>(0.0006) | -0.0016**<br>(0.0006) | -0.0039*<br>(0.0016) | -0.0032*<br>(0.0016) |
| Neither agree or disagree | -0.0058**<br>(0.0021) | -0.0064**<br>(0.0021) | -0.0136*<br>(0.0053) | -0.0105*<br>(0.0052) |
| Somewhat agree | -0.0106**<br>(0.0038) | -0.0113**<br>(0.0037) | -0.0007*<br>(0.0004) | -0.0006<br>(0.0003) |
| Strongly agree | 0.0200**<br>(0.0071) | 0.0218**<br>(0.0070) | -0.0004<br>(0.0002) | -0.0003<br>(0.0002) |
