# Supplementary material for: Institutional trust, scientific literacy, and information sources: What factors determine people's attitudes toward COVID-19 vaccines of different origins in China?
Source: Front Public Health. 2023 Feb 20;11:1092425. doi: 10.3389/fpubh.2023.1092425 (PMC9986272; doi:10.3389/fpubh.2023.1092425)
Supplement: Supplementary file 4 [file Table_4.pdf]

Table 4: The marginal effect of scientist trust (Model7-Model10)

	Scientist trust			
	Model7	Model8	Model9	Model10
Strongly disagree	-0.0047**<br>(0.0009)	-0.0044**<br>(0.0008)	-0.0321***<br>(0.0052)	0.0257***<br>(0.0050)
Somewhat disagree	-0.0028*<br>(0.0006)	-0.0027**<br>(0.0006)	-0.0068***<br>(0.0012)	-0.0056***<br>(0.0011)
Neither agree or disagree	-0.0118**<br>(0.0018)	-0.0111**<br>(0.0018)	-0.0234***<br>(0.0038)	-0.0185***<br>(0.0037)
Somewhat agree	-0.0216**<br>(0.0409)	-0.0197**<br>(0.0030)	-0.0012**<br>(0.0004)	-0.0010**<br>(0.0004)
Strongly agree	0.0200**<br>(0.0071)	0.0379**<br>(0.0053)	0.0007*<br>(0.003)	-0.0006*<br>(0.0003)
